# Dr. Fe Del Mundo: The Pioneer Who Transformed Pediatrics and Child Healthcare in the Philippines

**DOI:** 10.7759/cureus.68109

**Published:** 2024-08-29

**Authors:** Joselv E Albano, Janelle Lara G Mirhan, Anushree Bansal, Dheeraj Jayakumar

**Affiliations:** 1 Neurosurgery, Southern Philippines Medical Center, Davao City, PHL; 2 Obstetrics and Gynecology, Brokenshire Medical Center, Davao City, PHL; 3 Pediatrics, Davao Medical School Foundation, Inc., Davao City, PHL

**Keywords:** female physician, fe del mundo, medical history, philippines, pediatrics, historical vignette

## Abstract

Dr. Fe del Mundo was a female Filipino pediatrician, humanitarian, and advocate of Filipino children, dedicating her life to seven decades of service. Her programs on maternal and child health, preventive medicine, nutrition, vaccination, and family planning transformed the healthcare landscape in the Philippines. Overcoming adversity, she pursued a road less traveled for women of her time. After training in the United States of America, she returned to the Philippines to take care of sick children during the Second World War and later opened the first pediatric hospital in the country. Her research, inventions, and health programs set the groundwork for the improvement of pediatric and childhood outcomes in the country and the world. She is a woman of many firsts, being a beacon for generations of physicians and women. Her legacy endures through the programs she started, the lives she has saved, the doctors she trained, and the institutions she has founded for the welfare of the children she dearly cared for.

## Introduction and background

**Figure 1:**
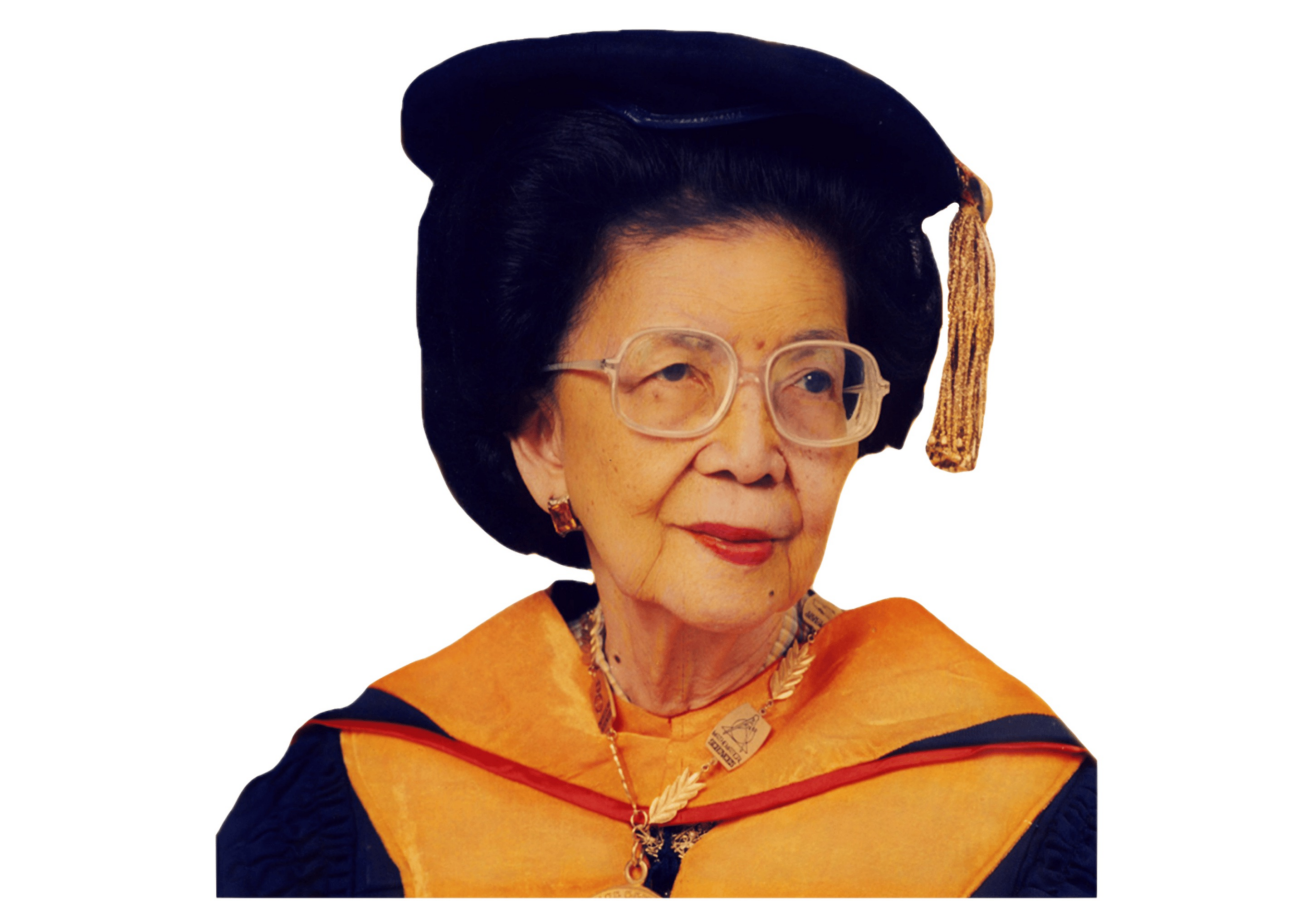
Dr. Fe Del Mundo Image Credit: National Academy of Science and Technology (Philippines) [1]; Fair Use

Dr. Fe del Mundo (1911-2011) is the Philippines’ most widely regarded pediatrician who not only transformed the landscape of pediatrics but also the overall healthcare system in the country (Figure 1) [1]. She has inspired countless women of every generation to pursue the medical field and strive for excellence. She shattered stereotypes with her decades of service and was known not only for her skills as a pediatrician but also for the humanitarian legacy she left behind. She became a symbol of dedication and service, healing generations of children under her care and paving the way for the future for the hundreds of doctors she trained. This review presents the storied life of Dr. del Mundo as the pioneer of pediatrics in the Philippines.

## Review

Early life and educational background

Dr. Fe Del Mundo was born on November 27, 1911, in Manila, Philippines. She was the sixth among her eight siblings [2]. Her early life was marked by numerous adversities. Of her eight siblings, three passed away in infancy, and at the age of 11, her older sister died of appendicitis. She also lost her mother shortly after graduating from high school. The passing of Fe’s younger sister Elisa, who had expressed a wish to work as a doctor for the underprivileged, encouraged her to pursue a career in medicine [3]. Her sister's death was said to have been the stimulus for Fe’s determination to pursue medicine [4]. She began her education at the University of the Philippines, Manila, and received her Associate in Arts degree in 1928, setting the groundwork for her future studies. Despite financial constraints, she pursued her Doctor of Medicine program and graduated as valedictorian in a class of 70 in 1933 from the University of the Philippines, demonstrating her remarkable academic abilities [5]. Upon graduation, she also received the award for the "Most Outstanding Scholar in Medicine" by the Colegio Medico-Farmaceutico de Filipinas [2]. 

The poor standards of child healthcare at that time in the Philippines, with many children dying of preventable diseases, and her personal experience of the lack of knowledge on this subject pushed her to become a pediatrician. The then president of the Philippines, Manuel Quezon, due to her outstanding academic record, offered her a scholarship for further training at any school of her choice in the United States of America where she spent five years advancing her knowledge [2,3]. She underwent a two-year research fellowship at the Harvard Medical School and earned a master’s degree in Bacteriology from Boston University in 1940. She rotated, studied, and did research on pediatrics and infectious diseases in different institutions including Mount Sinai Hospital, Columbia University, University of Chicago's Billings Hospital, Johns Hopkins Hospital, and Boston Children’s Hospital. She also took a public health class at the Massachusetts Institute of Technology [2,4,6]. Despite several attractive offers for her to establish a professional career in the United States, she returned home to the Philippines in 1941 [4].

Transforming pediatrics in the Philippines 

Fe returned to the Philippines right at the beginning of the Japanese invasion of the country in 1941. Although initially wanting to get a government teaching position, she volunteered in the Red Cross taking care of sick children in the internment camps, and set up a hospice at the University of Santo Tomas. Her efforts and care eventually led to her being known as the “Angel of Santo Tomas” [7-9]. When the Japanese closed down the hospice in 1943, she transferred and became the director of the North General Hospital (now Jose R. Reyes Memorial Medical Center) in Manila. The hospital, though initially intended as a children’s hospital, was eventually converted to an emergency unit to attend to civilian casualties of the Battle of Manila, then later as a charity hospital. She is recognized as the country’s first woman to be director of a government general hospital [2,7]. It was during her stint as a director that a School of Nursing was opened in 1945 to augment the nursing services in the area and the country [2]. She later joined the University of Santo Tomas faculty and the Far Eastern University, being head of the Department of Pediatrics for 20 years [4].

In 1957, after selling her home and properties, she opened the Children’s Memorial Hospital (later renamed Fe del Mundo Medical Center) which at that time was the Philippines’ first pediatric hospital [4,8]. She is said to have lived on the second floor of the hospital, working around the clock to help as many children as she could even until her last years of life [7]. She improved pediatric care at the municipal and local levels by linking her hospital with health workers in those communities. In 1962, she started sending rehydration teams to rural communities to treat diarrhea with many volunteers later joining the initiative. Through her leadership, the Institute of Maternal and Child Health was established in 1966, the first of its kind in Asia, furthering pediatric care in rural areas. They established numerous clinics and trained thousands of personnel to provide improved services in remote areas and promote community programs on disease prevention, nutrition, vaccinations, and family planning [2]. Her community-oriented approach incorporated local traditions such as "hilot" into primary healthcare through her work with midwives and family planning, as well as the use of locally available solutions for medicine [2,8]. Examples include her promotion of the BRAT (banana, rice, apple, tea) diet in the treatment of diarrhea which saved countless lives worldwide [10]. She is also credited for inventing a bamboo incubator using two layers of locally accessible bamboo baskets lined with heated water bottles allowing babies in rural areas to be incubated without requiring any electricity [4]. Her tireless efforts elevated the delivery of pediatric healthcare nationwide, a turnaround from the dismal standards of her youth.

**Figure 2:**
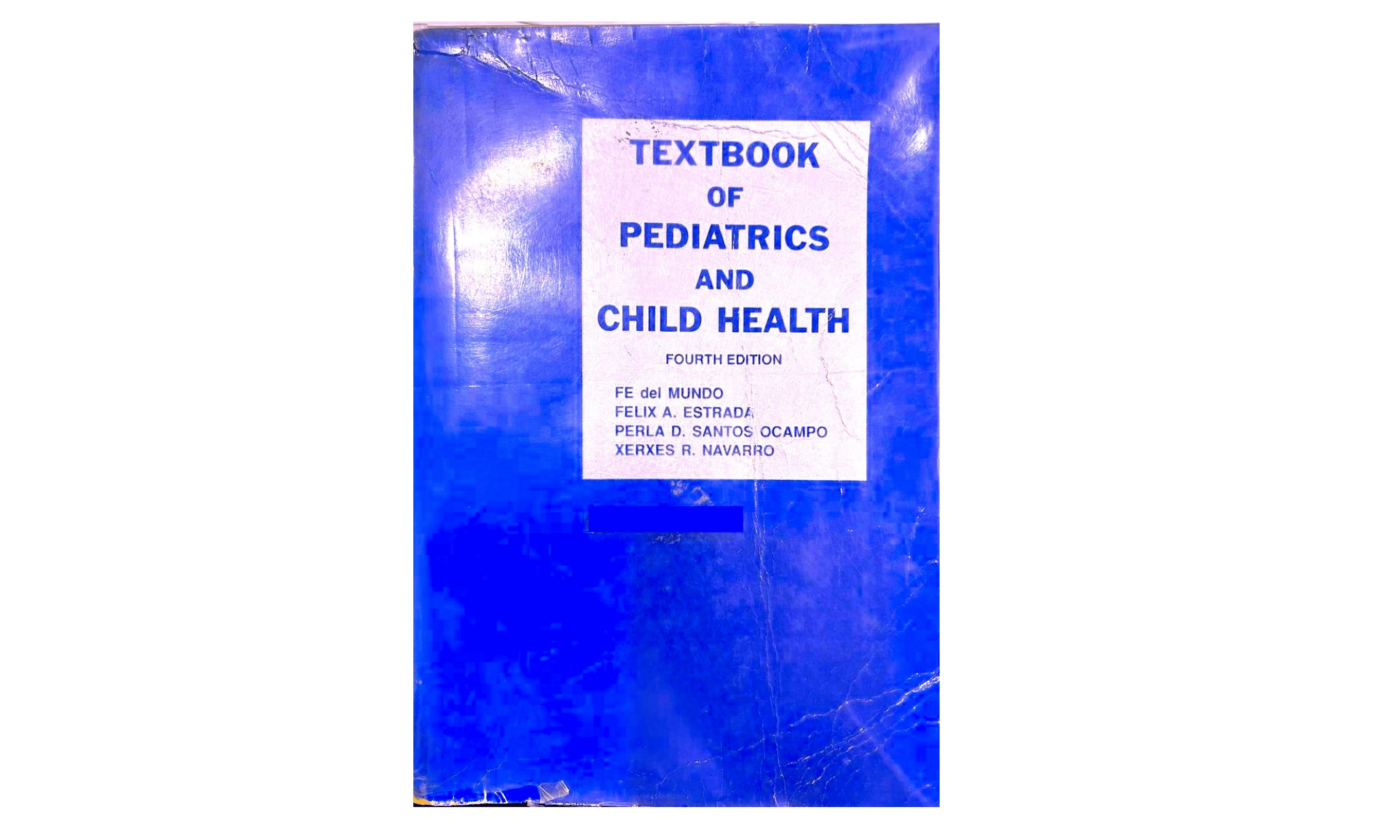
Textbook of Pediatrics and Child Health (4th Edition) Image Source: The Davao Medical School Foundation-College of Medicine; used with permission

Aside from taking on pediatric residents to train under her, she also published the Philippines’ first textbook on pediatrics in 1976 (Figure 2) [11]. With many subsequent editions published, this book has been taught across medical schools in the country and has helped train many generations of physicians. She furthered the field through her research and contributions to pediatrics, also becoming a consultant for the World Health Organization for pediatric care [2]. With over 100 scientific papers, she carried out research on communicable diseases in children setting the groundwork for the vaccination and tuberculosis programs in the country. Her initiatives on vaccination and disease prevention became a model for many countries in the Southeast Asia region and the world [5,8]. Always trying to reach out to the masses, she also authored a weekly column in the Manila Sunday Times promoting maternal and child health [12].

An inspiration to women in medicine 

In addition to being the first woman in the country to serve as the director of a government general hospital and founding the nation’s first pediatric hospital, Dr. Fe del Mundo continues to stand out as a trailblazer who overcame the many challenges of her generation. In 1947, she was the first Filipino diplomate certified by the American Board of Pediatrics. In 1949, she founded and was the first president of the Philippine Medical Women’s Association. In 1952, she became the first female president of the Philippine Pediatric Society, in 1962, the first Asian president of the Medical Women’s International Association, in 1969, the first female president of the Philippine Medical Association, and in 1980, the country’s first female national scientist [2,13-16]. Her accolades did not end there, winning numerous awards and recognition for her work, service, and contributions to humanity. Some of the most notable include the Elizabeth Blackwell Award for Outstanding Service to Mankind in 1966, the Most Outstanding Pediatrician and Humanitarian award at the 15th International Congress of Pediatrics, the Ramon Magsaysay Award for Public Service in 1977, recognition as a National Scientist in 1980, the Most Outstanding Physician Award by the Philippine Medical Association in 2002, the Gusi Peace Prize for her advocacy on pediatric health in 2003, the Blessed Teresa of Calcutta Award from the Alfonso Yuchengco Foundation in 2008, and the Grand Cross (Bayani) of the Order of Lakandula by the Philippine president in 2010 [2,5,8,10,17,18].

It is clear that not only did she advance healthcare in her part of the world but she also served as a beacon to many women of her time and generations to come to pursue a career in the medical field. She was known to have encouraged women to take up medicine saying that women make good pediatricians [4]. She inspired generation after generation of women, showing that the change that can be accomplished in a lifetime can be astronomical.

Legacy 

She lived till the age of 99 and served until her death in 2011. She continued to live in the hospital true to her eight decades of commitment and service. She did not marry or have children, choosing instead to be the mother of thousands of Filipino children. She dedicated herself to improving the lives of so many children and the state of healthcare in the country, even giving up her own possessions for this pursuit. Today, the Fe del Mundo Medical Center still endures, keeping on with her call to the service of humanity. She left the world a better place, having trained new physicians and empowered the local community to ensure that children can enjoy longer and healthier lives. Her humanitarian efforts paved the way for a healthier Filipino generation, in which her legacy lives on forever. 

## Conclusions

Dr. Fe del Mundo’s life and work demonstrate her fervent service towards the goal of improving the lives of children across the country and the world. She overcame many hurdles to advocate for the children that she fought and cared for till her death. Her efforts transformed the pediatric landscape in the national and global stage. Her advocacies on prevention, nutrition, vaccinations, and family planning penetrated the rural areas of the country elevating the state of healthcare for families nationwide. She broke stereotypes doing anything she can to promote the welfare of the patients she cared for. Her work set the path towards a healthier country as she helped shape almost a century of medicine in the Philippines. Because of her tireless dedication, children today can live and enjoy longer and happier lives.
